# Haplotype-Resolution Transcriptome Analysis Reveals Important Responsive Gene Modules and Allele-Specific Expression Contributions under Continuous Salt and Drought in *Camellia sinensis*

**DOI:** 10.3390/genes14071417

**Published:** 2023-07-08

**Authors:** Qing Zhang, Ziqi Ye, Yinghao Wang, Xingtan Zhang, Weilong Kong

**Affiliations:** 1 Shenzhen Branch, Guangdong Laboratory for Lingnan Modern Agriculture, Genome Analysis Laboratory of the Ministry of Agriculture, Agricultural Genomics Institute at Shenzhen, Chinese Academy of Agricultural Sciences, Shenzhen 518120, China; zhangqing970@126.com (Q.Z.); leavesziqi@163.com (Z.Y.); zhangxingtan@cass.cn (X.Z.); 2 Center for Genomics and Biotechnology, Haixia Institute of Science and Technology, Fujian Agriculture and Forestry University, Fuzhou 350002, China; wangyinghao98520@163.com

**Keywords:** *Camellia sinensis*, transcriptome, salt and drought stress, co-expression network, allele-specific expression

## Abstract

The tea plant, *Camellia sinensis* (L.) O. Kuntze, is one of the most important beverage crops with significant economic and cultural value. Global climate change and population growth have led to increased salt and drought stress, negatively affecting tea yield and quality. The response mechanism of tea plants to these stresses remains poorly understood due to the lack of reference genome-based transcriptional descriptions. This study presents a high-quality genome-based transcriptome dynamic analysis of *C. sinensis*’ response to salt and drought stress. A total of 2244 upregulated and 2164 downregulated genes were identified under salt and drought stress compared to the control sample. Most of the differentially expression genes (DEGs) were found to involve divergent regulation processes at different time points under stress. Some shared up- and downregulated DEGs related to secondary metabolic and photosynthetic processes, respectively. Weighted gene co-expression network analysis (WGCNA) revealed six co-expression modules significantly positively correlated with *C. sinensis’* response to salt or drought stress. The MEpurple module indicated crosstalk between the two stresses related to ubiquitination and the phenylpropanoid metabolic regulation process. We identified 1969 salt-responsive and 1887 drought-responsive allele-specific expression (ASE) genes in *C. sinensis*. Further comparison between these ASE genes and tea plant heterosis-related genes suggests that heterosis likely contributes to the adversity and stress resistance of *C. sinensis*. This work offers new insight into the underlying mechanisms of *C. sinensis’* response to salt and drought stress and supports the improved breeding of tea plants with enhanced salt and drought tolerance.

## 1. Introduction

Crop plants are often challenged by two of the most widespread abiotic stresses: salt stress and drought stress, which have resulted in yield reductions that have perplexed plant biologists over the past few decades. Predictions indicate that by the year 2050, salinity and drought will cause the serious salinization of over 50% of all arable land, which poses a significant challenge for food security to meet the demands of the growing population [1,2]. Therefore, it is crucial to understand the underlying mechanisms of salinity and drought tolerance in plants to breed species that possess strong resistance and adaptability.

Plants respond to diverse abiotic stresses through different mechanisms that are regulated at multiple levels. Salt stress affects plants by causing early occurring osmotic stress and subsequent ionic stress, which can disturb cellular osmotic and ion homeostasis, resulting in ion toxicity due to the accumulation of chloride (Cl^−^) and sodium (Na^+^) in the cytoplasm. This can hinder the normal growth of plants [3,4,5,6]. Drought stress is perceived by plants as osmotic stress caused by water deficiency, which restricts aboveground growth by changing photosynthetic efficiency and altering root architecture and resource allocation to avoid dehydration [7,8]. In addition to physiological responses, a series of complex regulatory networks are executed in plants at the molecular and protein levels to sense and respond to these stresses [9,10]. These regulatory networks usually involve the participation of crucial second messengers, including reactive oxygen species (ROS) signaling [11,12], calcium signaling (Ca^2+^) [13], and protein phosphorylation [9], which affect the expression of downstream genes by altering their homeostasis. For example, downregulating the expression level of *OsGRS17* in rice improves drought stress tolerance by modulating ROS accumulation and stomatal closure [14], while in tomatoes, the ectopic expression of *AtGRXS17* can improve heat tolerance by reducing ROS accumulation [15]. Protein phosphorylation is also a common and critical signal in response to various abiotic stress conditions. Many protein phosphorylation-related genes, including *SnRK2s*, *RLKs*, and *MAPKs*, are activated by the binding of ABA or RAF (Raf-like kinase) to their receptor proteins to regulate various downstream proteins [16], such as transcription factors, to respond to abiotic stress in Arabidopsis, maize [17], and rice [18]. Unlike protein phosphorylation, Ca^2+^ signaling is perceived by the Salt Overly Sensitive (SOS) pathway and leads to the exportation of the Na^+^ from root epidermal cells and xylem parenchyma cells in response to salt stress [10,13]. Although the mechanisms of response to salt and drought stress have been well studied in model plants such as Arabidopsis and rice [9], the co-expression networks toward continuous drought and salt stress are poorly understood in important economic crops.

Allele-specific expression (ASE) refers to the expression divergence of the gene alleles inherited from parents due to transcriptional or epigenetic regulation. It is one of the important genetic factors leading to phenotypic variation and adaption to different environments [19]. In mammals, ASE has traditionally been associated with X-chromosome inactivation and genomic imprinting in autosomal genes. ASE has also been demonstrated in plants, including Arabidopsis, maize, rice, and apple [20,21]. Several studies have suggested that ASE plays a vital role in response to abiotic stress. For example, ASE in selfing species such as barley and tomato has been found to be related to developmental variation and drought stress [19,22]. Interestingly, a recent study demonstrated that the ASE response to drought stress in apples is triggered by the methylation of a MITE insertion in the promoter of one allele [21]. These results indicate that the ASE could be a potentially important resource for improved breeding of abiotic resistance. However, ASE related to the response to abiotic stress in *Camellia sinensis*, a self-incompatible species, is poorly understood.

The tea plant (*Camellia sinensis* (L.) O. Kuntze) is one of the most popular non-alcoholic beverages crops worldwide, inherited for thousands of years due to its unique taste and contribution to health benefits [23]. In many areas, the tea plant has suffered from serious abiotic stress, including salt, drought, and cold, which adversely impact the quality and production of tea. Therefore, a deep understanding of the mechanisms of the tea plant’s response to the abiotic stress is vital for industrial upgrading of tea. Recently, several transcriptomic analyses have revealed the potential mechanisms of *C. sinensis’* response to chilling [24,25], drought [26,27], and salt [28]. Some cross-talking signals have indicated the existence of interactions between different abiotic stresses [27]. For example, the tolerance for cold, salt, and drought stress can be enhanced by exogenous melatonin via improving antioxidant defense in tea plants [29]. However, the complex molecular regulatory mechanisms underlying the response to abiotic stress, especially salt and drought stress, remain mostly unknown in tea plants due to the almost non-existent reference genome-based analyses in previous studies.

The decoding of the tea plant genome [30,31,32,33], especially the haplotype-resolved TGY (Tieguanyin, *C. sinensis* var. *sinensis*) genome [34], provides an opportunity to analyze the potential regulatory mechanisms underlying the response to salt and drought stress at the allele gene level in tea plants. To understand the potential mechanisms of salt and drought stress in tea plants, we conducted differential expression and co-expression analyses based on the TGY genome using our previously generated transcriptomic data in response to continuous salt and drought stress [35]. Importantly, we also characterized the extent of ASE and its interaction with salt and drought stress in tea plants at the whole-transcriptome scale. Our results provide insight into the underlying mechanisms of tea plants in response to salt and drought stress and contribute to the improved breeding of tea plants in the future.

## 2. Materials and Methods

### 2.1. Transcriptome Data Preparation

The RNA-seq data used in this research were generated in a previous study [35] and were downloaded from the European Nucleotide Archive database under project accession number PRJEB11522. The details of the sample treatment were described in the previous study [35]. Briefly, one-year-old *C. sinensis* cv. Tieguanyin plants with similar growth states and sizes were used as cuttings and treated with a 1/2 dose Hoagland nutrient solution for three days. The cultured cuttings were divided into three groups, with one group continuing to be cultured with the 1/2 dose Hoagland nutrient solution for 0, 24, 48, and 72 h, respectively, marking the control group, while the other two groups were transferred to the 1/2 dose Hoagland nutrient solution with 200 mM NaCl or 25% polyethylene glycol (PEG) for 24, 48, and 72 h, respectively. Fresh leaf samples (second and third leaves under the bud) were collected from five individuals at each time point of treatment (NaCl and PEG) or from the control group, snap-frozen with liquid nitrogen, and stored at −80 °C for further processing. The total RNA within each sample was isolated using the CTAB method as described by Shi et al. (2007) [36]. The samples with an RNA Integrity Number (RIN) ≥ 7 were selected through the RNA integrity testing by Agilent 2100 Bioanalyzer (Agilent, Santa Clara, CA, USA). Equal amounts of total RNA from five individuals of each time point of NaCl, PEG stress, and controls were mixed for cDNA library construction. The ten paired-end cDNA libraries were constructed as described by Zhang et al. (2017) [35], and all well-prepared cDNA libraries were sequenced on the Illumina HiSeq™ 2500 platform at the Center for Genomics and Biotechnology of Fujian Agriculture and Forestry University (Fuzhou, China).

### 2.2. Data Procession and Gene Expression Calculation

The raw data were initially processed using Trimmomatic (v0.36) [37] to remove adapter sequences and low-quality sequences with the following parameter: “LEADING:3 TRAILING:3 SLIDINGWINDOW:4:15 MINLEN:50”. The resulting clean reads were mapped to the monoploidy *C. sinensis* TGY reference genomes [34] using HISAT2 (v2.20) [38]. The SAM format files produced were batch-converted to BAM files and sorted with SAMtools (v1.11) [39]. Finally, all sorted BAM files were used as input for StringTie (v2.1.2) [40] to calculate the fragment per kilobase of transcript per million mapped reads (FPKM) value for each sample.

### 2.3. Differential Gene Expression Analysis

The count of mapped reads in each sample against the genes of TGY genome was determined using featureCounts [41]. Then, an in-house PYTHON script was used to construct the reads count matrix. To identify differential expression genes (DEGs) in multiple dimensions, a multifactor design was used to differentiate gene expression changes due to time points, different stress, or the interaction between the two, using a pairwise comparison approach to identify the DEGs. The DEG sets were identified using the EdgeR packages [42] with the parameters of false discovery rate (FDR)-adjusted *p*.value = 0.05 and square-root-dispersion = 0.1.

### 2.4. Allele-Specific Expression (ASE) Analysis

The clean reads from the ten samples were mapped to the haplotype-resolved TGY genome [34] using HISAT2 [38]. The output file was processed and FPKM was calculated for each allele genes using the pipeline described in Section 2.2. The count of mapped reads against the alleles of haplotype-resolved TGY genome was determined using featureCounts [41], and ASEs were identified using in-house PYTHON scripts based on the criterion of twofold changed expression between the pair of gene alleles.

### 2.5. Functional Enrichment Analysis

The gene functions were annotated using eggnog-mapper [43] against the eggNOG database (http://eggnog5.embl.de/#/app/home; accessed on 1 July 2022) with defeat parameters. Gene Ontology (GO: http://www.geneontology.org; accessed on 1 July 2022) and Kyoto Encyclopedia of Genes and Genomes (KEGG: http://www.genome.jp/kegg/; accessed on 1 July 2022) enrichment analyses were conducted using the R package ClusterProfile (v4.0.3) [44]. Briefly, the gene sets of interest were applied to detect overrepresented GO terms or KEGG pathway against the universal set of genes using Fisher’s exact test. The Benjamini–Hochberg method [45] was used for the multiple testing adjusting of *p*-values, and the FDR-adjusted *p* < 0.05 was considered significant for GO terms or KEGG pathways.

### 2.6. Weighted Gene Coexpression Network Analysis

The expression matrix was constructed using in-house PYTHON scripts, with FPKM values of each sample normalized and calculated by StringTie [40]. Co-expression network analysis was performed using the R package weighted gene co-expression network analysis (WGCNA) [46]. After removing genes with low expression (FPKM < 5 in all samples) across samples, co-expression modules were generated with default settings; however, the minModuleSize was set to 80, and the CutHeight was set to 0.3. The Cytoscape [47] was used for network visualization, with the parameters of |GS| > 0.2 and |MM| > 0.8.

## 3. Results

### 3.1. RNA-Seq Analysis of Continuous Salt and Drought Stress Tolerance in Tea Plants

To comprehensively explore the underlying mechanisms of *C. sinensis* in response to salt and drought stress, transcriptome sequencing data from 10 samples, including continuous salt and drought stress as well as controls for 0, 24, 48, and 72 h, were obtained from the previous study [35]. After adapter and low-quality reads filtering, an average of 79,792,118 clean reads were acquired in each sample and mapped against the monoploid and haplotype-resolved TGY reference genomes, respectively (Table S1). The proportion of clean reads mapped to the haplotype-resolve TGY genome (90.17% (NaCl_24h) ~96.04% (PEG_24h)) was higher than the proportion mapped to the monoploid TGY genome (87.57% (NaCl_24h) ~93.27% (PEG_24h)) (Table S1), indicating the reliable quality of the clean data for further analysis.

**Figure 1:**
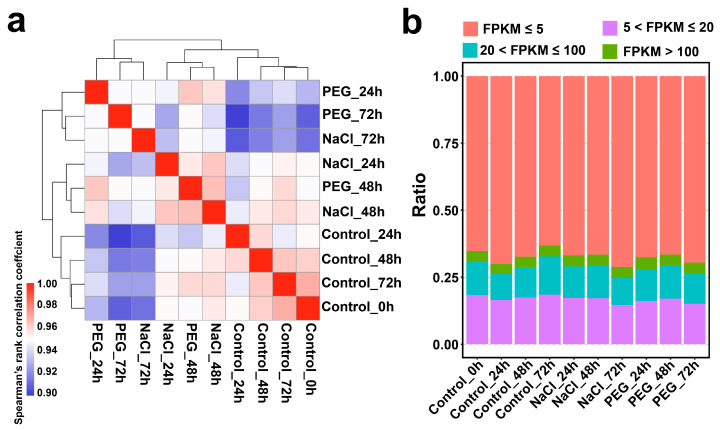
The transcriptome feature of *C. sinensis*′ response to continuous salt and drought stress. (**a**) All samples were clustered based on the filtered gene profile with FPKM > 1 using the Spearman correlation coefficient. (**b**) The proportion of gene expression was counted with four scales (FPKM ≤ 5, 5 < FPKM ≤ 20, 20 < FPKM ≤ 100, and FPKM > 100) in all samples.

To explore potential correlations between the expression patterns of samples under different stress conditions, a Spearman correlation coefficient analysis of the expression of all samples was conducted using the FPKM matrix (Figure 1). The results showed that the gene expression of tea plant seedling under drought stress at 24 h clustered together with those at 72 h of drought and salt stress, while the expression patterns of salt stress at 24 and 48 h and drought stress at 48 h were clustered in another group (Figure 1a). This suggests that *C. sinensis* exhibits divergent expression patterns in response to salt and drought stress at 24 h but displays similar expression patterns in response to salt and drought stress at 48 and 72 h (Figure 1a). In addition, the expression pattern of *C. sinensis* under the nutrient solution culture at 0, 24, 48, and 72 h was grouped in one group (Figure 1a), indicating little change in the expression pattern of *C. sinensis* in the control group.

We next classified genes according to their expression levels in each tested sample as low-expressed, medium-low-expressed, medium-expressed, and high-expressed genes (Figure 1b). The proportions of these four types of genes were similar in all tested samples. In general, the proportion of low-expressed genes was higher than that of medium-low-expressed and medium-expressed genes (Figure 1b), and the proportion of high-expressed genes was the lowest. This suggests that although gene expression in *C. sinensis* changes during the stress process, the overall change tends to maintain a dynamic balance.

### 3.2. The Dynamic Change of Tea Plant Transcriptome in Response to Salt and Drought Stress

**Figure 2:**
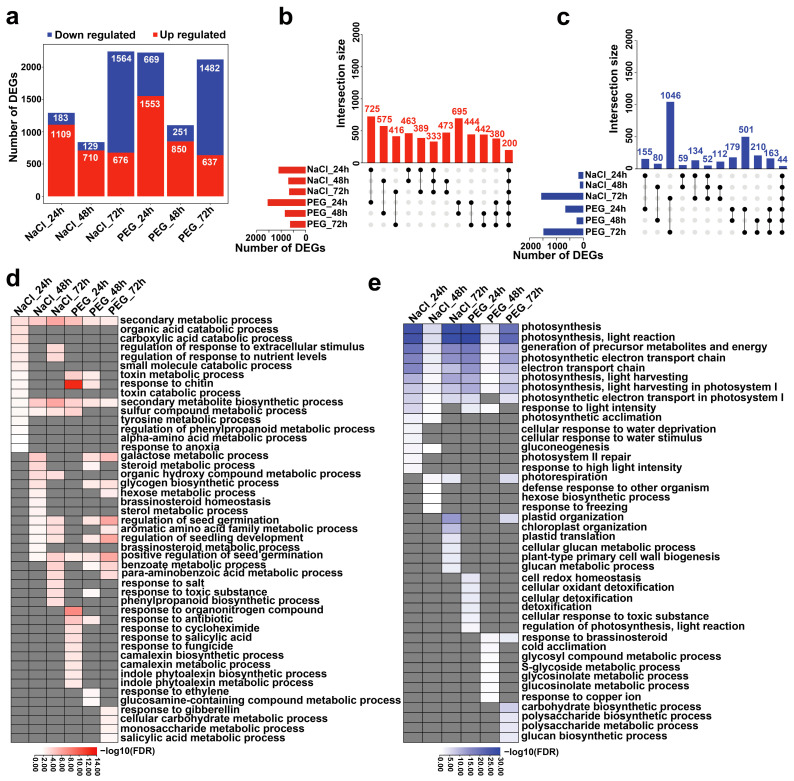
The transcriptomic divergent and dynamic changes in *C. sinensis’* response to salt and drought stress. (**a**) The histogram illustrates the total number of the DEGs of each treated sample compared with the corresponding control. (**b**,**c**) Boxplots of upregulated (**b**) and downregulated (**c**) DEGs and the overlap in or between salt and drought stress. The top histogram shows the number of DEGs for each overlapping combination as indicated by the connected circles below, and the bottom left horizontal histogram displays the total number of DEGs identified in each treated sample. (**d**,**e**) The heatmap shows the GO enrichment results of upregulated and downregulated DEGs in *C. sinensis* under each time point treatment of salt and drought stress. The top 20 non-redundant and significant GO terms of *C. sinensis* under each time point treatment of salt and stress are shown. The color intensity in both heatmaps indicates −log10 transformed ‘adjusted *p*-values,’ where darker red/blue indicates a more significant enrichment, and gray indicates missing enrichment.

To characterize the expression patterns of genes under salt and drought stresses at dynamic time points, we performed a multifactor comparison (time points + treatments + time points: treatments) to identify differentially expressed genes (DEGs). A total of 2244 upregulated and 2164 downregulated genes were identified in samples under salt and drought stresses compared with the control (Figure 2). The GO enrichment analysis revealed that upregulated genes were mainly enriched in the plant’s response to the stimulus processed, whereas downregulated genes were primarily involved in metabolic processes associated with life activities (Figure S1).

We further performed pairwise comparisons between treated samples and the control for each associated time point to investigate the dynamic change of *C. sinensis’* transcriptome in response to stresses. The number of DEGs in *C. sinensis’* response to salt and drought stress at each time point ranged from 839 to 2240. NaCl_72 h, PEG_72 h, and PEG_24 h had the most DEGs compared with other time points under both stresses, accounting for ~69% of the total DEGs, while the lowest number of DEGs was identified at 48 h of salt or drought stress (Figure 2a). The number of upregulated genes identified in the samples at 24 h and 48 h under both stresses was higher than that of downregulated genes, while the number of upregulated genes was lower than that of down-regulated genes at 72 h under both stresses (Figure 2a). The intersection analysis among the three time points of *C. sinensis’* response to salt or drought stress showed that the shared number of upregulated genes across all tested time points under salt stress (333) was similar to that under drought stress (380), and the number of upregulated genes shared between at 24 h and 48 h (463 in NaCl, 695 in PEG) was higher than that at any other two time points (Figure 2b). Moreover, the shared number of downregulated genes in response to salt and drought stress was highest at 24 h and 72 h (Figure 2c). In addition, the comparison of upregulated and downregulated genes at the consistent time points between *C. sinensis’* response to salt and drought stresses showed that the number of upregulated genes was the highest at 24 h and the lowest at 72 h, while the number of downregulated genes was the highest at 72 h and the lowest at 48 h (Figure 2b,c), suggesting that *C. sinensis* had complex gene expression regulation patterns in response to salt stress and drought stress.

To investigate the biological functions associated with DEGs identified in *C. sinensis’* response to salt and drought stress, we conducted GO enrichment analysis for upregulated and downregulated genes at each time point of salt and drought stress. The results showed that up- and downregulated genes had stress- and time-point-specific enriched GO terms (Figure 2d,e). For example, upregulated genes in *C. sinensis’* response to NaCl_24 h were enriched in organic acid catabolic-related processes, while those at other time points, such as at the “carboxylic acid catabolic process”, “α-amino acid metabolic process”, and “tyrosine metabolic process”, were enriched. Upregulated genes in response to PEG_24 h were cataloged in hormone signal-mediated processes, including the “response to salicylic acid”, “indole phytoalexin biosynthetic process”, and “response to organonitrogen compound”. Interestingly, the GO term of the secondary metabolic process and photosynthesis were enriched in up- and downregulated genes, respectively, across all time points of both salt and drought stress (Figure 2d,e). This is consistent with the results of KEGG pathway analysis (Figure S2), suggesting a potential relationship between these processes in *C. sinensis’* response to salt and drought stress.

### 3.3. Co-Expression Gene Network Analysis Uncovers the Similar or Divergent Responsiveness to Salt and Drought Stress

**Figure 3:**
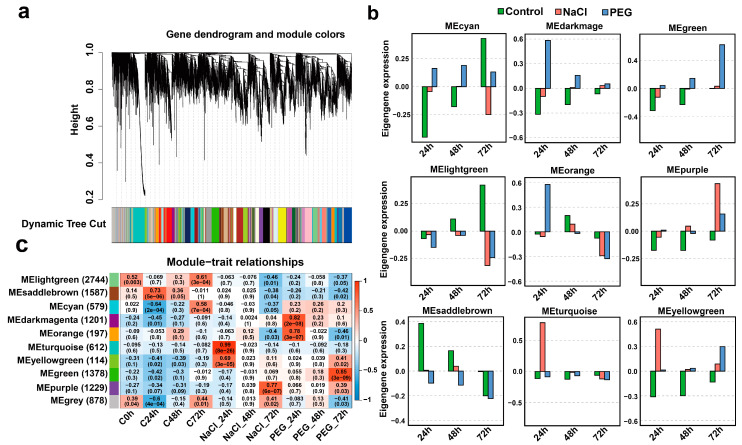
WGCNA co-expression network analysis for the dynamic transcriptome of *C. sinensis’* response to salt and drought stress. (**a**). Hierarchical cluster tree exhibiting co-expression modules identified by WGCNA. The major tree branches constitute 10 modules labeled by different colors, and module ‘Grey’ represents unassigned genes. (**b**). The barplots indicate the expression of module eigengenes. (**c**). Heatmap representing the Pearson correlation between different modules. The eigengenes and all samples are indicated by rows and columns, respectively.

To further investigate the dynamic transcriptional organization underlying salt and drought responses and the divergence between the response process of these two types of stress, we conducted WGCNA analysis on all samples at different time points under salt and drought stress (Figure 3 and Figure S3). A total of nine network modules were identified, containing numbers of co-expression genes ranging from 114 to 2744. All network modules can be clustered into three groups, one with four modules and the other two with three and two modules, respectively (Figure S3), indicating a possible relationship between different modules on expression regulation. Furthermore, six of nine network modules showed significant correlations with at least one time point of salt or drought stress (Figure 3c and Figure S4). We analyzed the expression patterns of each network module to detect the ones that most correlated with salt or drought stress. Among the nine modules, the expression of genes in two modules (MEdarkmagenta and Meorange) was the highest during drought stress at 24 h, and in another three modules (Meyellowgreen, Megreen, and Mepurple), gene expression was the highest at 72 h (Figure 3b). Similarly, the gene expression of MEturquoise and MEyellowgreen was highest during salt stress treatment at 24 h, while that of MEpurple was highest at 72 h, and these modules showed a high positive correlation with the time point under the stress treatment and high expression (Figure 3b). This suggests a complex regulatory network involved in *C. sinensis’* response to salt and drought stress processes. Moreover, both the MEyellowgreen and MEpurple modules were significantly positively related to drought stress and salt stress, and their GO terms were primarily enriched in secondary metabolism processes (Figure S5), indicating that these two modules are probably the important bridge connecting the regulation network between *C. sinensis’* response to salt and drought stress. Notably, all the modules showed low expression levels and non-significant correlation at 48 h under the two types of stress (Figure 3b,c).

**Figure 4:**
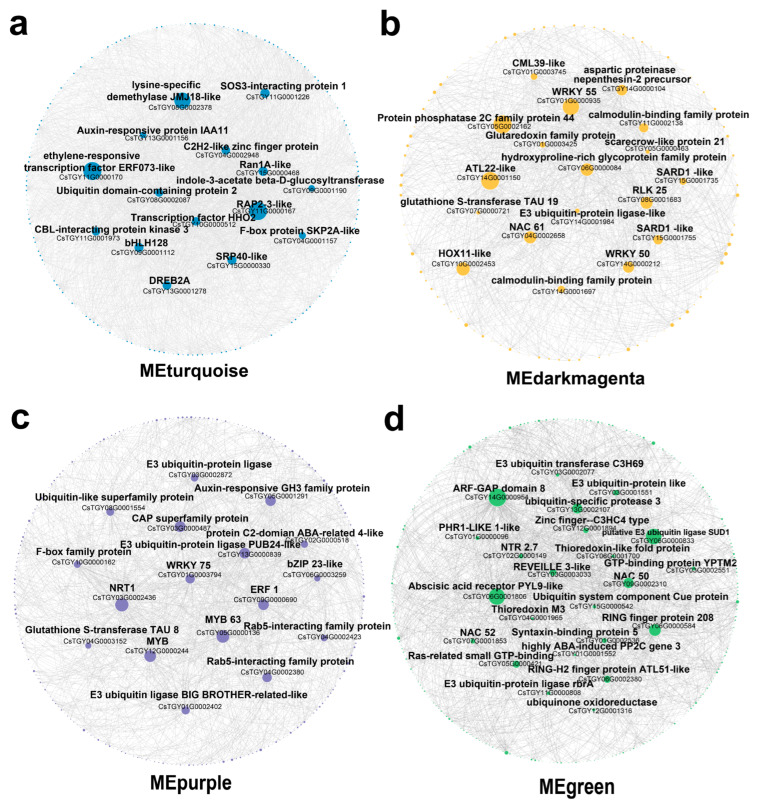
The Cytoscape visualization of four co-expression network modules. The correlation network of the (**a**) MEturquois, (**b**) MEdarkmagenta, (**c**) MEpurple, and (**d**) MEgreen modules were represented by the co-expression genes with an edge weight ≥ 0.3. The size of the circle indicates the number of edges of the gene, and all hub genes were labeled with the gene ID and gene name around the circle.

To identify the potential candidate genes in response to salt and drought stress in *C. sinensis*, we further characterized the network modules that significantly positively correlated with salt or drought stress. The Cytoscape exhibition enabled us to identify a total of 15, 18, 17, 22, and 16 hub genes that harbored the most edges with other genes among the modules of MEturquoise, MEdarmagenta, MEpurple, MEgreen, and MEorange respectively, based on the screen of high weight value (Figure 4 and Figure S6). Most of these hub genes are related to functions that encode key transcription factors, such as *WRKY*, *bHLH*, *MYB*, *NAC*, and *DREB*, and vital enzymes, such as *RLK* and *CBL* (Figure 4). These results indicate a complex transcriptional regulation involved in *C. sinensis’* response to salt and drought stress.

### 3.4. Stress-Responsive Allele-Specific Expression (ASE) Is Interesting in Heterozygous Diploid

**Figure 5:**
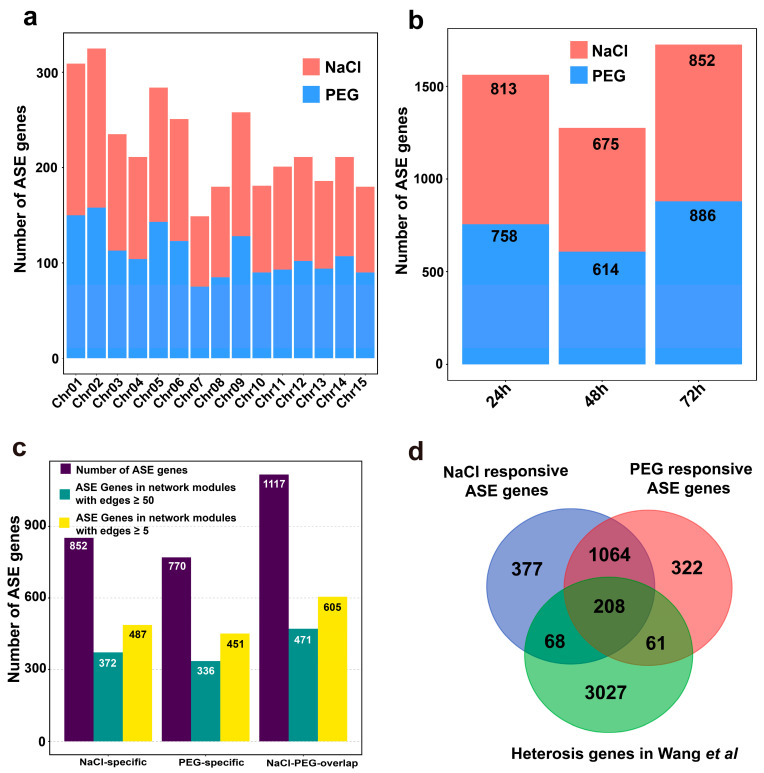
The ASE genes categorization in *C. sinensis’* response to salt and drought stress. (**a**) The boxplot illustrates the distribution of salt- or drought-responsive ASE genes across *C. sinensis* genome. The *x*-axis indicates the 15 chromosomes, and the *y*-axis indicates number of ASE genes. (**b**) The count of ASE genes that responded to salt or drought stress at 24, 48, and 72 h, respectively. (**c**) The boxplot shows the ASE of salt- and drought-specific genes, as well as the intersection of these two stresses that represents in network modules with high connection (with edges ≥5 and ≥50). (**d**) The Venn diagram displays the intersection between salt-, drought-responsive, and heterosis-related ASE genes [49].

Allele-specific expression (ASE) genes are widely found in heterozygous diploids and allopolyploids [22,48], and they play an important role in most life processes. To investigate whether and how ASEs participate in *C. sinensis’* response to salt stress and drought stress, we identified the ASEs in *C. sinensis* using the dynamic transcriptome of treatment with salt stress and drought stress at 24, 48, and 72 h in this study. A total of 1717 salt- and 1655 drought-responsive ASE genes were identified, suggesting that ASE genes widely exist in *C. sinensis* (Figure 5 and Supplementary Data 1). The salt- or drought-responsive ASE genes distributed ranged from 149 (74 salt-responsive and 75 drought-responsive) to 325 (167 salt-responsive and 158 drought-responsive) on the 15 chromosomes (Figure 5a). These genes were located slightly higher on chromosomes 1 and 2 than the other chromosomes in *C. sinensis*, which suggests that these ASE genes were distributed without chromosomal bias. We further investigated the ASE genes across the dynamic process by treatment with salt stress and drought stress, respectively. The number of ASE genes identified in 24 h (813 salt-responsive and 758 drought-responsive) and 72 h (852 salt-responsive and 886 drought-responsive) was higher than that at 48 h (Figure 5b), indicating that ASE genes probably play a divergent role at different time points under salt stress and drought stress.

For genes displaying stress-responsive ASE, we performed a pairwise comparison for the change of bias between the control and treated conditions (Table 1). We identified that 37.98% (1128), 30.60% (936), and 39.42% (1186) of ASE genes exhibited responsiveness to salt stress or drought stress at 24 h, 48 h, and 72 h, respectively (Table 1 and Figure S7), suggesting that ASE genes widely participate in the response to salt stress and drought stress. The number of ASE genes specific to response to salt stress at 24 h and 48 h was higher than that to drought stress, while the number of ASE genes at 72 h of salt stress was fewer than that of drought stress (Figure S7). Compared to stress-specific ASE genes, most of the ASE genes in *C. sinensis* were both salt- and drought-responsive. For example, 46.54% (552) of ASE genes at 72 h showed both stress responses, which was higher than that at 24 h (39.27%, 443) and 48 h (37.71%, 353) (Table 1 and Figure S7). These results suggest a potential relationship between ASE genes involved in salt and drought stress. In addition, 57.16%, 58.57%, and 54.16% of ASE genes represented in salt stress-, drought stress-specific, and shared within both kinds of stress, respectively, were highly connected (with a number of WGCNA edges ≥ 5) in the network modules (Figure 5c), suggesting that ASE genes likely play an important role in the response to salt stress and drought stress.

## 4. Discussion

Salt stress and drought stress are two of the most prevalent stresses in plants, and they pose increasing environmental problems that significantly impact crop yield and food security. Although the molecular mechanism of plant response to salt and drought stress has been extensively studied in some model plants, such as *Arabidopsis* and rice [10], it has received little attention in some important crops, particularly tea plants. As one of the most important beverage crops worldwide, the tea plant has been subjected to abiotic stress for a long time, especially salt stress and drought stress, which have caused substantial economic losses to the tea industry. Hence, studying the molecular mechanism of tea plant response to abiotic stress is of great significance. Previously, Wan et al. [28] identified that some DEGs were mainly involved in Ca^2+^ signal transduction, the ABA pathway, and MAPK cascades from the transcriptome of tea plant responding to salt stress. In our previous study, we identified 3936 and 3715 DEGs at all time points of salt stress and drought stress, respectively [35]. However, these analyses were still limited by the lack of available high-quality and complete reference genomes at that time. Transcriptome de novo-based analyses, on the one hand, have incomplete transcript information. On the other hand, the allelic redundancy of some genes, especially in highly heterozygous genomes such as tea plants, can cause quantitative deviation of gene expression and affect the analysis of the entire transcriptome. In this study, we first used haploid reference genomes to analyze the transcriptome of tea plants in response to salt and drought stress [34]. This approach accurately reflects these dynamic processes and allows for a more comprehensive exploration of the key genes involved in these regulatory processes.

### 4.1. C. sinensis Exhibit a Divergent Global Transcriptional Response to Salt and Drought Stress

Salt stress and drought stress tolerance are complex traits that involve numerous genes and key pathways. These two types of stress cause different physical and physiological damage to plants. Although both stresses cause osmotic stress, drought stress can also cause ionic toxicity. Previous studies have shown that plants are more sensitive to PEG-mediated drought stress than salt stress [50]. In this study, we found that the number of DEGs under drought stress was much higher than that under salt stress at 24 h. This suggests that tea plants are more sensitive to drought stress than salt stress. This may be because osmotic stress caused by drought stress can cause immediate physiological responses, such as stomatal closure and reduced transpiration [51], while plant response to salt stress involves the slower process of ion absorption and accumulation in the roots [52].

To adapt to salt stress and drought stress, plants have developed diverse stress-responsive signaling pathways and activated defense mechanisms [53]. Our study revealed that *C. sinensis* displayed divergent global transcriptional responses to salt stress and drought stress, which were evidenced by the low intersection of the downregulated genes within the comparison groups except for NaCl_72 h versus PEG_72 h. The high ratio of up- (57.45%) and downregulated (66.67%) GO terms exhibited at time-point andstress-specific points also indicate the divergent function implication and responding mechanism executed in *C. sinensis’* response to salt and drought stress even at the same time point.

The WGCNA packages [46] were widely applied in transcriptome analysis to explore core genes related to key traits or stress-tolerant and, ultimately, to build the potential regulation network. In this study, the WGCNA analysis revealed that the co-expression networks of *C. sinensis* were probably relatively independent in the early stage of the response to salt and drought stress since *C. sinensis’* response to salt stress and drought stress at 24 h was most significantly correlated with two different co-expression modules (MEturquoise, *p* = 8 × 10^−26^; MEdarkmagenta, *p* = 2 × 10^−8^), respectively, and KEGG enrichment showed that the co-expression genes of the MEturquoise module were mainly involved in polysaccharide and amino acid metabolism-related pathways. Although sugar and amino acid metabolism, especially proline, have been widely reported to play important roles in response to salt and drought stress [54], we did not find any pathways related to these secondary metabolites during 24 h of drought stress (Figure S5), which was presumably due to the spatiotemporal expression differences of genes involved in these pathways in the early stage of response to salt stress and drought stress. Moreover, the most weighted hub genes in these two modules showed no obvious homologous hub genes intersection found between MEturquoise and Medarkmagenta, which also evidenced the notion that *C. sinensis* exhibit a divergent global transcriptional response to salt and drought stress at the early stage. For example, the hub genes identified in the Medarkmagenta modules were mainly involved in the Ca^2+^ dependent kinase signal transduction pathway, such as *PP2C44*, *ATL22*, and *RLK25*, as well as the genes that encoded some important transcript factors, including *WRKY55*, *WRKY50*, *NAC61*, and *HOX11*. The *WRKY55* and *WRKY50* genes were reported to be induced in soybean plants under drought stress [55], and the former can positively regulate leaf senescence and the defense response through ROS and salicylic acid (SA)-related pathways in Arabidopsis [56]. In addition to drought stress, the homeobox-leucine zipper protein *HOX11* has been reported to be involved in a variety of abiotic stress responses [57,58]. Thus, these transcript factors possibly play a key role in *C. sinensis’* response to early drought stress. In contrast, the hub genes of MEturquoise were mainly related to hormone response pathways such as *ERF073-like*, *RAP2-3-like*, Auxin-responsive protein *IAA11*, and *indole-3-acetate β-D-glucosyltransferase*, as well as transcription factors such as *bHLH128*, *HHO2*, *C2H2*, and *DREB2A*. Most of these genes have been reported to participate in abiotic stress in other species [59,60,61]; for example, *RAP2-3-like* also has been noted to induce significantly high expression in wheat under 24 h of salt stress [62]. Another ethylene-responsive transcription factor, *ERF073-like*, has been reported to be involved in drought stress response in chickpeas [63], but no studies have found its correlation with salt stress response, suggesting that this would be a novel and vital candidate gene to study in *C. sinensis*.

### 4.2. The Crosstalk between C. sinensis Response to Salt and Drought Stress

Crosstalk is a mechanism that plants have developed over a long period of evolution to cope with various stresses and abiotic factors in the natural environment. Many regulators, such as Ca^2+^, ROS, MAP-kinase, hormones, and secondary metabolites, are believed to be involved in crosstalk to assist plants in coping with various stresses [64,65,66]. In this study, 1098 upregulated and 1134 downregulated DEGs (65.71% and 68.77% of the total, respectively) were identified as shared in *C. sinensis*’ response to salt and drought stress, GO enrichment showed that the secondary metabolites process was widely employed in *C. sinensis*’ response to salt and drought stress, suggesting that a strong crosstalk likely exists in the secondary metabolites process between these two conditions. For instance, phenylpropanoids are secondary metabolites derived from the phenylalanine carbon skeleton, which are engaged in the plant response to multiple abiotic stresses [67]. Many studies have shown that phenylpropanoids and brassinosteroid (BR) are intricately linked with ROS pathways [67]. We found many pathways related to the phenylpropanoids metabolism and oxidative stress that were significantly enriched in *C. sinensis*’ response to salt and drought stress at all time points. Hence, we speculate that scavenging ROS through the phenylpropanoids-involved pathway may be an important strategy for tea plants to improve their tolerance to salt and drought stress. The phenylpropanoids synthesis process is known to be regulated by brassinosteroids. Researchers found that the external application of 24-epibrassinolide (EBR) induced the synthesis of phenolic content in grapes [68], and tea tree treated with EBR affects the accumulation of theanine and polyphenols, which are important products of the phenylpropanoids pathway [69]. The study of crabapple revealed that phenylalanine ammonia-lyase (PAL) regulates the synthesis of phenylpropanoids, which are rate-limiting enzymes of the pathway [70]. We also found that five (*CsTGY04G0002917*, *CsTGY07G0002047*, *CsTGY10G0001336*, *CsTGY11G0000747*, and *CsTGY01G0000382*) of eight PAL genes of *C. sinensis* were upregulated by salt or drought stress, and three (*CsTGY07G0002047*, *CsTGY11G0000747*, and *CsTGY01G0000382*) of them were upregulated by both stresses, suggesting crosstalk within the phenylpropanoid pathway between *C. sinensis*’ response to salt and drought stress.

Post-translational modifications, including protein phosphorylation, methylation, acetylation, and ubiquitination, play vital roles in plant development and abiotic stress tolerance [71]. Ubiquitination is one of the most important modifications that has been widely studied, and the process of protein ubiquitination depends on the participation of ubiquitin enzymes, including the ubiquitin-activating enzyme (E1), ubiquitin-conjugating enzyme (E2), and ubiquitin ligase (E3). The E3 is the key enzyme that determines the specificity of protein ubiquitination by recognizing and binding to the distinct substrates [72]. In this study, we identified 35 E3, three E2, and one E1 gene with upregulated expression, suggesting that ubiquitination probably plays an important role in *C. sinensis*’ response to salt and drought stress. In Arabidopsis, the E3 has been noted to be localized in the plasma membrane and participates in regulating ROS accumulation to improve salt and drought tolerance in plants [73]. This process can also be modulated through ABA-independent pathways [74]. The WGCNA analysis showed that the MEpurple module was closely correlated with the response of *C. sinensis* to both salt (*p* = 6 × 10^−7^) and drought stress (*p* = 0.03) at 72 h. In total, 4 of the 17 hub genes encoded ubiquitin ligase (*CsTGY03G0002872*, *CsTGY08G0001554*, *CsTGY13G0000839*, and *CsTGY01G0002402*), and three (CsTGY05G0001291, CsTGY02G0000518, and CsTGY09G0000690) encoded hormone-responsive proteins, indicating crosstalk in the ubiquitination-related pathway between *C. sinensis*’s response to salt and drought stress. In addition, many hub genes that encoded TFs, including *MYB63*, *WRKY75*, and *bZIP23*, were identified in MEpurple. Most of these TFs, such as *WRKY75*, have been revealed to play an important role in abiotic stress tolerance in poplar [75], Arabidopsis [76], and peanut [77], suggesting that these TFs potentially play a vital regulatory role in the crosstalk network involved in *C. sinensis* under salt and drought stress tolerance.

### 4.3. The ASEs in C. sinensis Response to Salt and Drought Stress

ASE is an interesting phenomenon that has been widely studied in plants [78]. In allopolyploids, ASE is also known as homeolog expression bias (HEB). A large number of studies have shown that ASE in allopolyploids plays a crucial role in improving their stress tolerance [48]. For example, more AT- than CT-biases were found in allotetraploid rapeseed under abiotic stress (drought, cold, and heat) [79]. In diploids, ASE is one of the most important mechanisms of heterosis formation, which has greatly improved the productivity of many crops globally [80]. There is also increasing evidence that ASE in diploids is involved in plant adaptation to stress. For instance, Mboup et al. found that ASE plays a vital role in the stress adaptation of two wild tomato species, *Solanum peruvianum* and *S. chilense* [81]. Comparing ASE in the F1 hybrid to the allelic expression in both parental lines revealed that numerous genes identified in the leaf and fruit exhibited significant ASE-by-watering regime interactions in tomatoes [22]. The changed ratios of ASE were also found in barley hybrids under drought stress [19]. Here, we identified 1969 salt-responsive and 1887 drought-responsive ASE genes in *C. sinensis*, among which 1117 ASE genes were shared in both stress- and drought-responsive genes, similar to the number identified in tomato’s’ response to a mild water deficit [22] but fewer than the number identified in cotton under salt stress [48]. It is possible that ASE response patterns differ in various species. Moreover, these salt-responsive or drought-responsive ASE genes were not significantly enriched in any pathway, which led us to speculate whether these ASE genes were dispersed and involved in potential regulatory roles in different pathways. This inference was evident in the result that over 30% of the ASE genes were involved in the co-expression network with a high connection (with edges ≥ 5). Furthermore, the inheritance contribution of ASE-based heterosis was studied in *C. sinensis* with its hybrid progeny, and most of these ASE genes were revealed involved in the metabolite that contributes to the high quality of tea [49]. Our results showed that 16.07% (276) and 16.25% (269) of salt-responsive and drought-responsive ASE genes, respectively, could be found in the ASE gene set of heterosis of Wang et al. [49], suggesting that the salt and drought tolerance of *C. sinensis* probably correlates with heterosis and possibly affects tea quality under stress through the ASE mechanism to some extent. However, these speculations require more sophisticated experimental evidence, such as genetic populations and genetic modification [82,83].

In conclusion, our study revealed a divergent global transcriptional regulation and local crosstalk among *C. sinensis’* response to salt and drought stress and found that ASE possibly plays an important role in *C. sinensis* coping with these two kinds of stress. Our findings provide important theoretical direction for the molecular breeding of tea plants with better salt and drought tolerance.

## Figures and Tables

**Table 1:** The statistic of salt- and drought-responsive ASE genes.

Specific Expression Pattern	Control	NaCl	PEG	No. of Genes
24 h	Conserved bias	Conserved bias	Conserved bias	1842 (62.02%)
	Bias	Bias	No bias	130 (4.38%)
	Bias	No bias	No bias	247 (8.32%)
	Bias	No bias	Bias	149 (5.02%)
	No bias	Bias	No bias	221 (7.44%)
	No bias	Bias	Bias	196 (6.60%)
	No bias	No bias	Bias	185 (6.23%)
48 h	Conserved bias	Conserved bias	Conserved bias	2123 (69.40%)
	Bias	Bias	No bias	101 (3.30%)
	Bias	No bias	No bias	167 (5.46%)
	Bias	No bias	Bias	124 (4.05%)
	No bias	Bias	No bias	198 (6.47%)
	No bias	Bias	Bias	186 (6.08%)
	No bias	No bias	Bias	160 (5.23%)
72 h	Conserved bias	Conserved bias	Conserved bias	1823 (60.58%)
	Bias	Bias	No bias	172 (5.72%)
	Bias	No bias	No bias	389 (12.93%)
	Bias	No bias	Bias	106 (3.52%)
	No bias	Bias	No bias	194 (6.45%)
	No bias	Bias	Bias	163 (5.42%)
	No bias	No bias	Bias	162 (5.38%)

## Data Availability

All data was downloaded from the European Nucleotide Archive database under project accession number PRJEB11522.

## Supplementary Materials

The following supporting information can be downloaded at: https://www.mdpi.com/article/10.3390/genes14071417/s1, Figure S1: The GO enrichment of the 4408 unique DEGs identified in *C. sinensis* by treatment with salt and drought stress. a: 2244 up-regulated genes, b: 2164 down-regulated genes; Figure S2: The KEGG pathway analysis for the DEGs of *C. sinensis* response to salt and drought stress. The heatmap of the KEGG pathway enriched by up-regulated (a) and down-regulated DEGs (b). The color intensity in both heatmap indicates “-Log10 (adjusted *p*-values)”, where for white to red represents an increasingly degree of enrichment; Figure S3: WGCNA analysis for the dynamic transcriptome of *C. sinensis* response to salt and drought stress. (a) Cluster dendrogram showing global relationship between different co-expression modules. (b) The correlation analysis across all modules; Figure S4: The modules of interest with significant correlations. Red circles represents positive correlations, blue circles represents negative correlations, and grey circles represents non-significant correlations; Figure S5: The GO enrichment for the genes of each modules. The colors indicate the adjusted *p*-value of each enriched GO term, and the width of each bar indicates the number of genes enriched in the term; Figure S6: The Cytoscape visualization for ME module. The orange circles indicate the gene that involve in this module, and grey line indicates the edges of each gene. The hub genes are marked in the right; Figure S7: The count of ASE genes in *C. sinensis* response to salt and drought stress at 24 h, 48 h, and 72 h; Table S1: The statistic of RNA-seq Data; Supplementary Data 1: The expression profile of Salt- and Drought-responsive ASE genes. All in-house scripts used in this study can be download from https://zenodo.org/record/8093922.
