# Supplementary material for: Haplotype-Resolution Transcriptome Analysis Reveals Important Responsive Gene Modules and Allele-Specific Expression Contributions under Continuous Salt and Drought in Camellia sinensis
Source: Genes (Basel). 2023 Jul 8;14(7):1417. doi: 10.3390/genes14071417 (PMC10379978; doi:10.3390/genes14071417)
Supplement: Supplementary file 1 [file genes-14-01417-s001.zip › Supplementary_file.pdf]

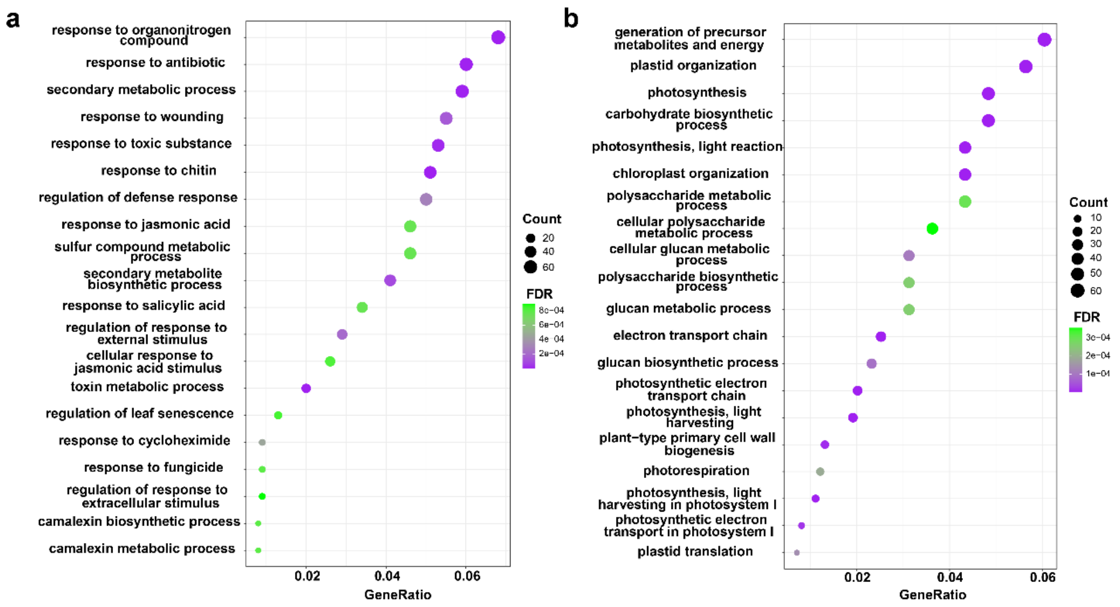

Figure S1. The GO enrichment of the 4,408 unique DEGs identified in *C. sinensis* by treatment with salt and drought stress. a: 2244 up-regulated genes, b: 2164 down-regulated genes.

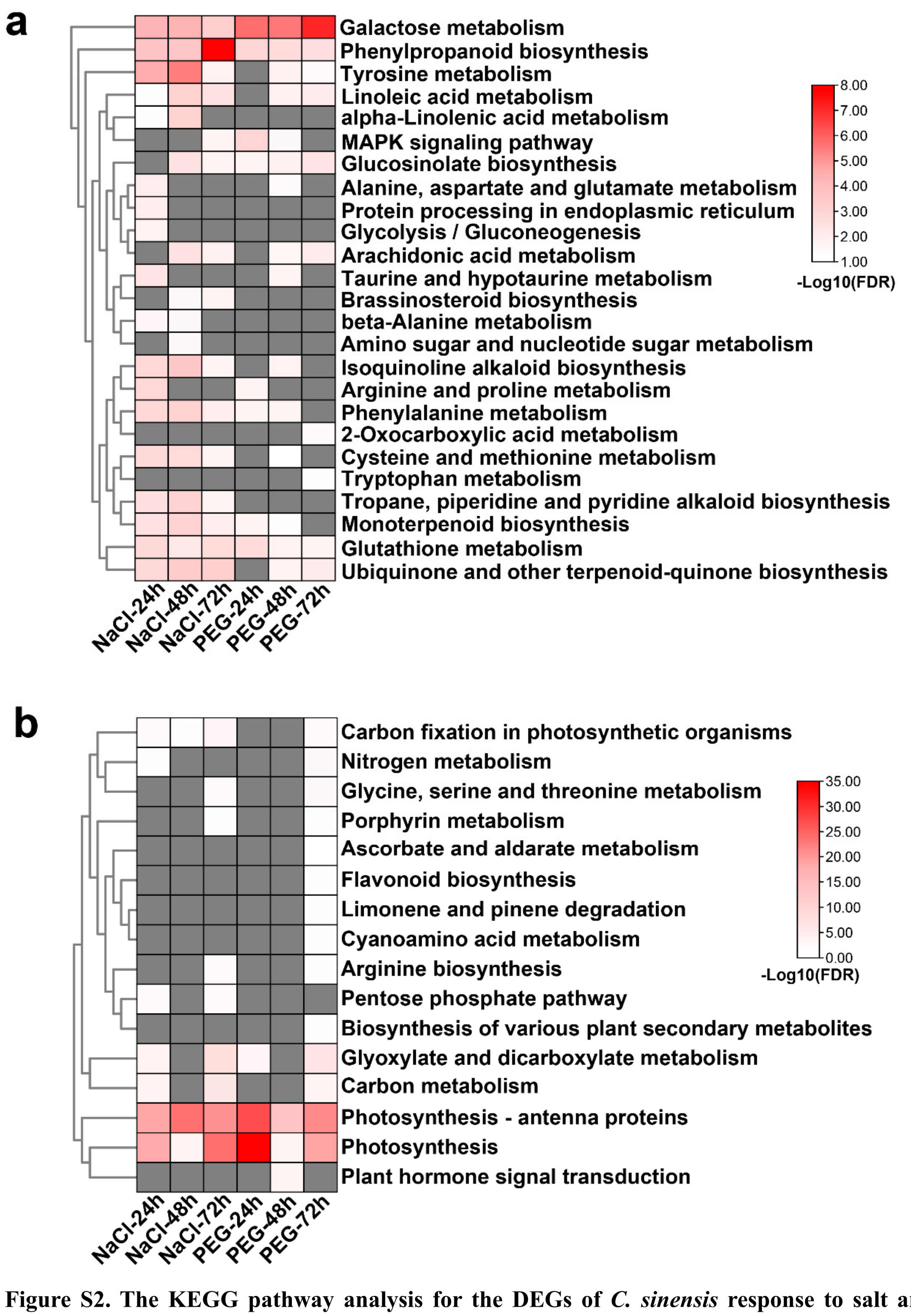

Figure S2. The KEGG pathway analysis for the DEGs of *C. sinensis* response to salt and drought stress. The heatmap of the KEGG pathway enriched by up-regulated (a) and down-regulated DEGs(b). The color intensity in both heatmap indicates "-Log10(adjusted p-values)", where for white to red represents an increasingly degree of enrichment.

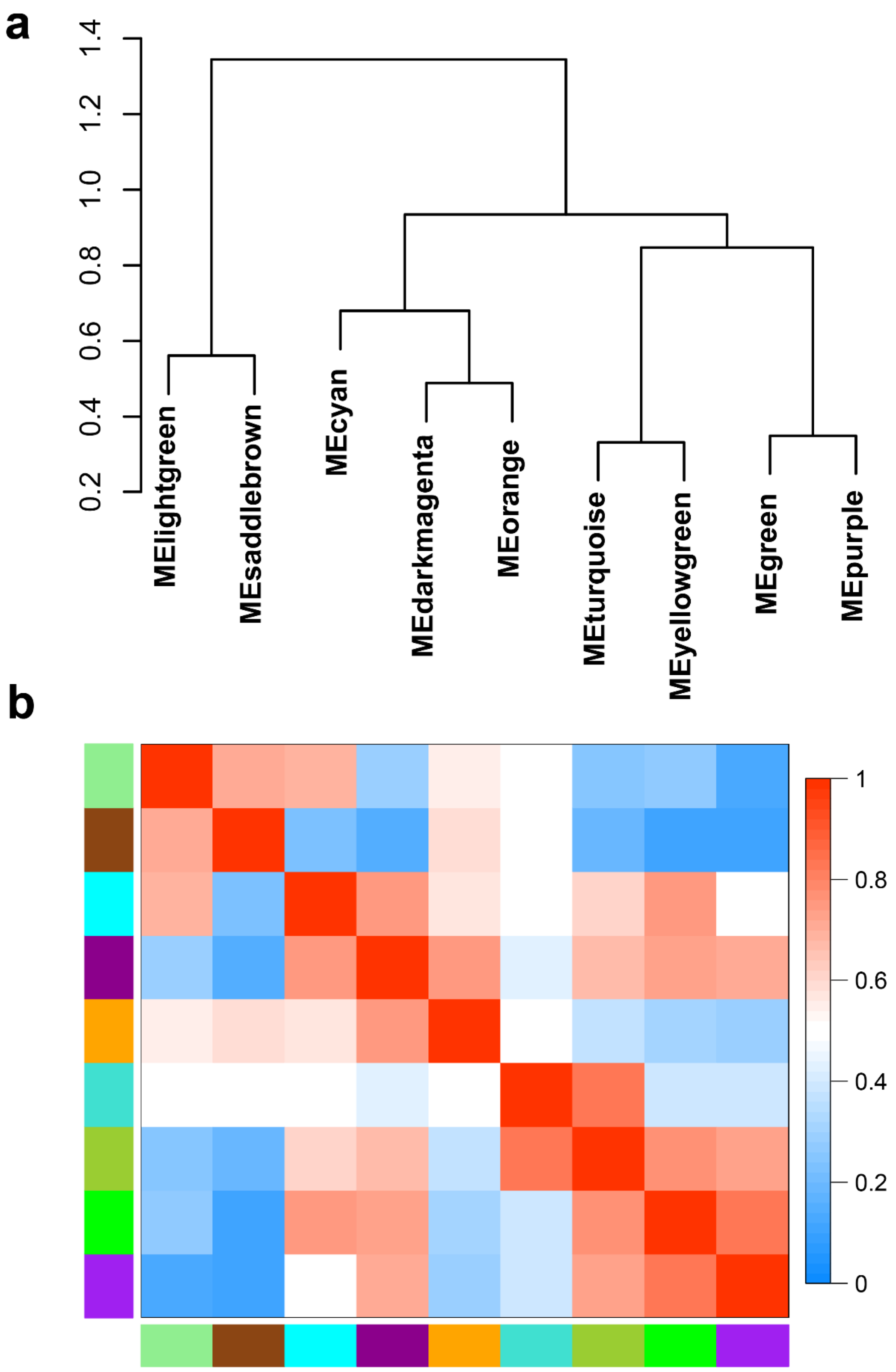

Figure S3. WGCNA analysis for the dynamic transcriptome of C. sinensis response to salt and drought stress. (a) Cluster dendrogram showing global relationship between different co-expression modules. (b) The correlation analysis across all modules.

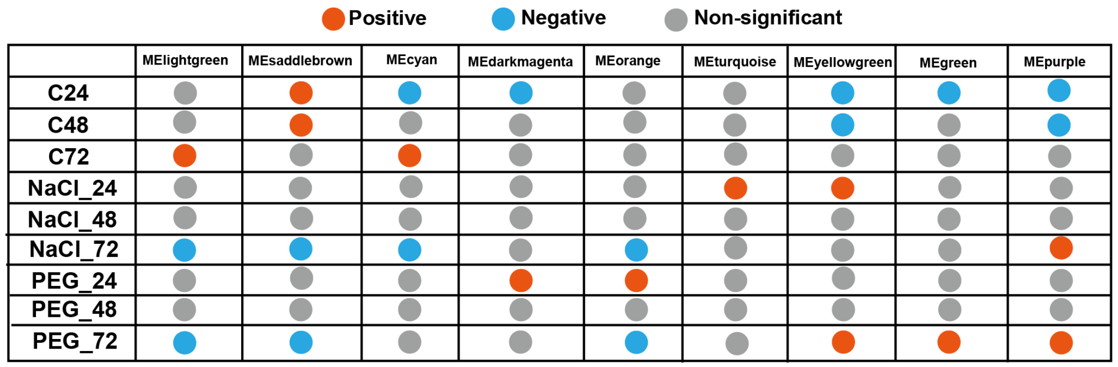

Figure S4. The modules of interest with significant correlations. Red circles represents positive correlations, blue circles represents negative correlations, and grey circles represents non-significant correlations.

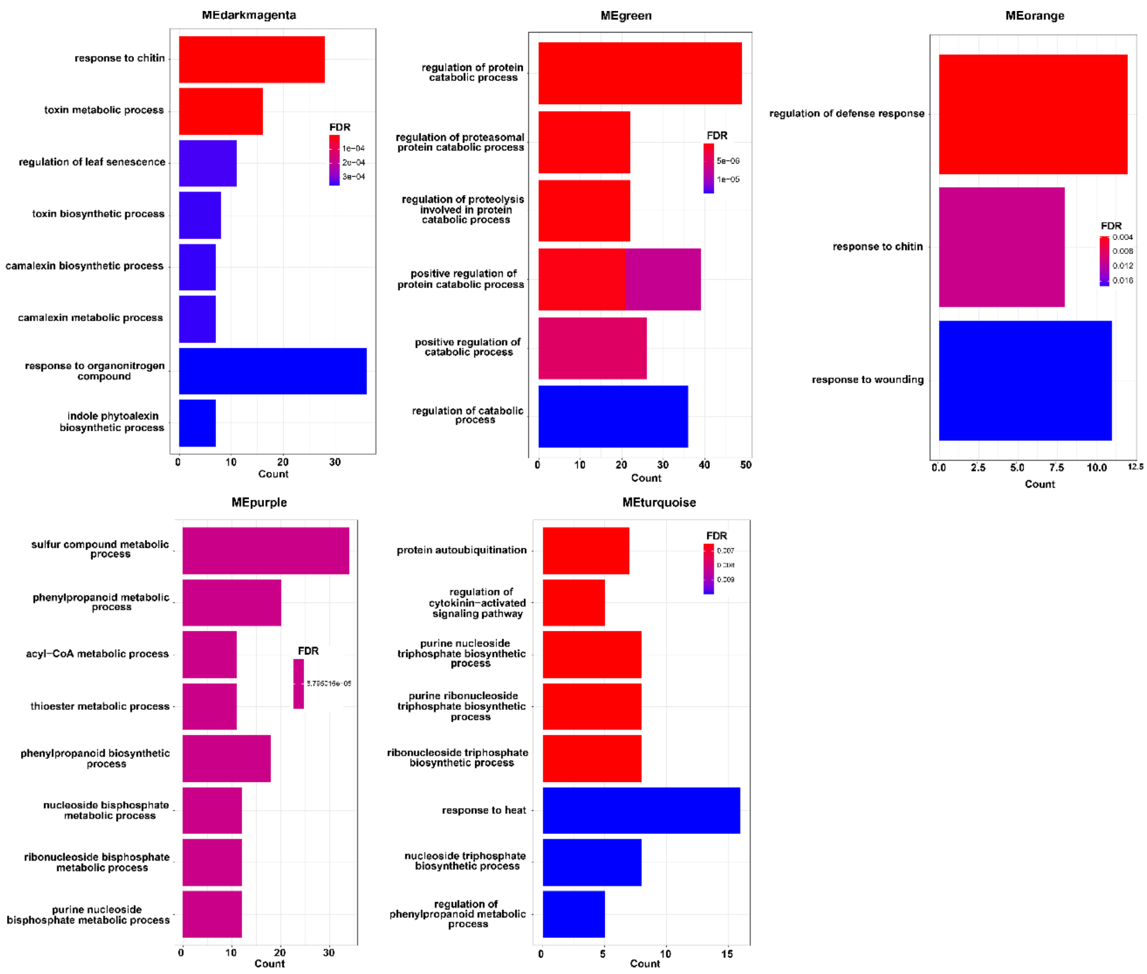

Figure S5. The GO enrichment for the genes of each module. The colors indicate the adjusted p-value of each enriched GO term, and the width of each bar indicates the number of genes enriched in the term.

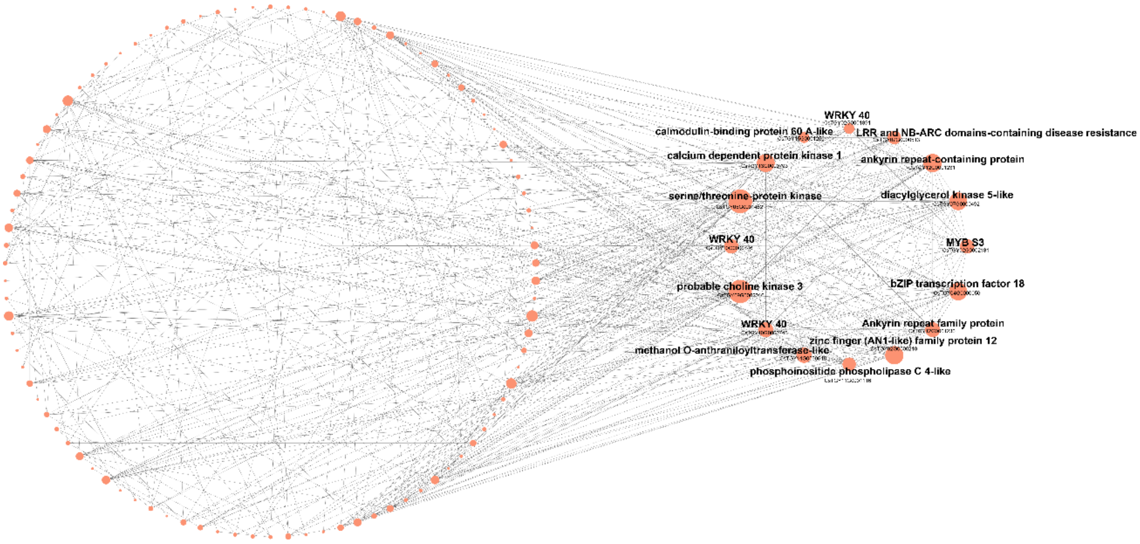

Figure S6. The Cytoscape visualization for ME module. The orange circles indicate the gene that involve in this module, and grey line indicates the edges of each gene. The hub genes are marked in the right.

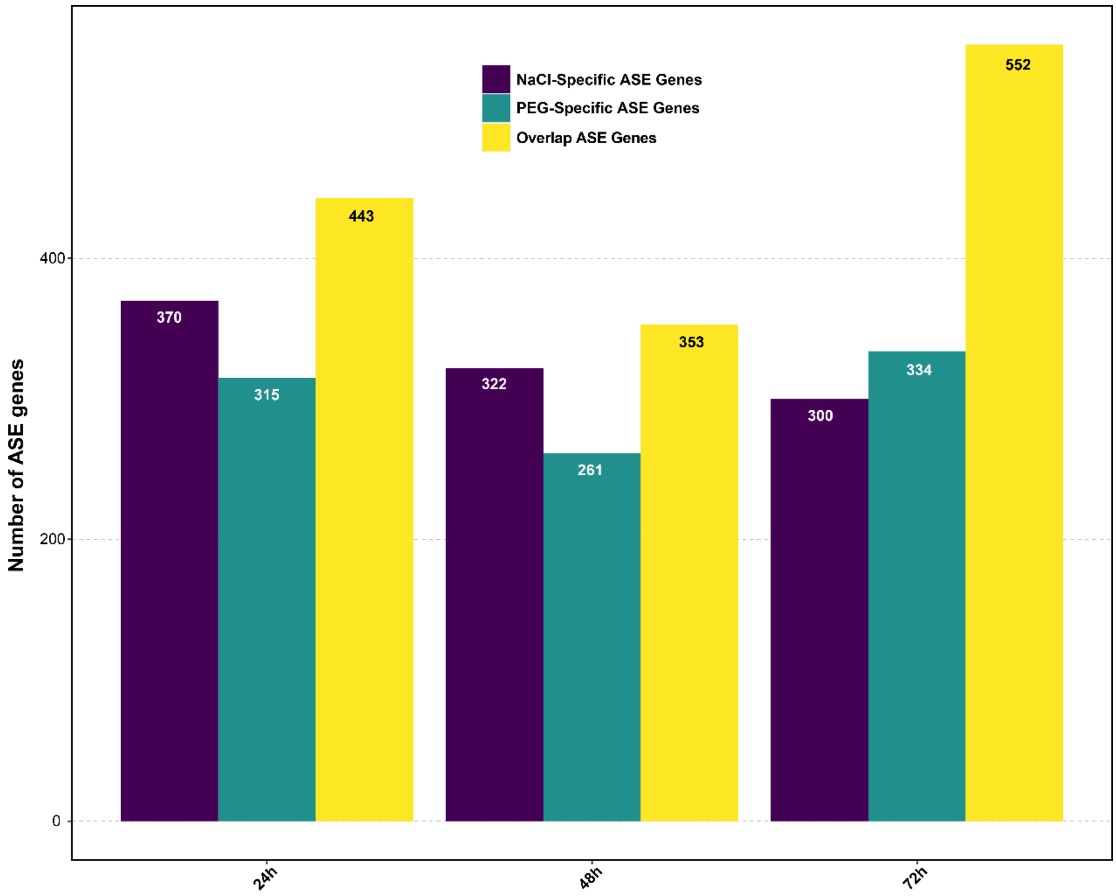

Figure S7. The count of ASE genes in *C. sinensis* response to salt and drought stress at 24h, 48h, and 72h.

Table S1. The statistic of RNA-seq Data.

**Samples**	**Number Raw reads**	**Number of clean reads**	**Ratio of mapping against *C. sinensis* TGY monoploidy genome**	**Ratio of mapping against *C. sinensis* TGY diploid genome**
**Control_0h**	38,845,398	36,201,112	92.98%	96.01%
**Control_24h**	28,291,358	26,310,952	93.12%	95.74%
**Control_48h**	45,006,622	41,786,164	92.37%	95.00%
**Control_72h**	28,515,952	26,468,918	92.40%	95.26%
**NaCl_24h**	56,014,018	51,955,674	87.57%	90.17%
**NaCl_48h**	46,719,712	43,177,880	91.12%	93.89%
**NaCl_72h**	46,747,596	43,669,424	92.38%	94.79%
**PEG_24h**	32,428,166	29,891,988	93.27%	96.04%
**PEG_48h**	39,224,568	35,805,180	92.72%	95.41%
**PEG_72h**	37,167,200	34,763,476	93.26%	95.62%
